# Impact of On-Demand Selective Suturing on Renal Function Preservation During Clampless Robotic-Assisted Partial Nephrectomy: Insights from a Large Multicentric Italian Cohort

**DOI:** 10.3390/jcm14217534

**Published:** 2025-10-24

**Authors:** Angelo Porreca, Davide De Marchi, Filippo Marino, Marco Giampaoli, Daniele D’Agostino, Francesca Simonetti, Antonio Amodeo, Paolo Corsi, Francesco Claps, Alessandro Crestani, Daniele Romagnoli, Pier Paolo Prontera, Gian Maria Busetto, Luca Di Gianfrancesco

**Affiliations:** 1 Department of Urology, Humanitas Gavazzeni, 24125 Bergamo, Italy; angelo.porreca@hunimed.eu (A.P.); davide.demarchi@gavazzeni.it (D.D.M.); marco.giampaoli@gavazzeni.it (M.G.); dott.dagostino@gmail.com (D.D.); francesca.simonetti@gavazzeni.it (F.S.); luca.digianfrancesco@gavazzeni.it (L.D.G.); 2 Department of Urology, Veneto Institute of Oncology, 35128 Padua, Italy; antonio.amodeo@iov.veneto.it (A.A.); paolo.corsi@iov.veneto.it (P.C.); francesco.claps@iov.veneto.it (F.C.); 3 Department of Urology, Azienda Sanitaria Universitaria Friuli Centrale, 33100 Udine, Italy; alessandro.crest@gmail.com; 4 Department of Urology, Policlinico Abano Terme, 35031 Abano Terme, Italy; infodottromagnoli@gmail.com; 5 Department of Urology, Ospedale SS. Annunziata, 74121 Taranto, Italy; pierpaolo.prontera@asl.taranto.it; 6 Department of Urology, Ospedali Riuniti Foggia, 71122 Foggia, Italy; gianmaria.busetto@unifg.it

**Keywords:** robotic-assisted partial nephrectomy, suturless, selective suturing, off-clamp, renal function preservation, oncologic control, high-volume centers

## Abstract

**Objectives**: To evaluate perioperative outcomes, renal function preservation, and short-term oncologic results of off-clamp, sutureless, or selectively sutured robotic-assisted partial nephrectomy (RAPN) in patients with renal tumors treated at multiple high-volume centers. **Methods**: This multicenter retrospective study included 250 patients who underwent off-clamp, sutureless/selectively sutured RAPN between January 2018 and December 2024. Patients with solitary kidneys, tumors > 7 cm, or prior renal surgery were excluded. All procedures were performed without renal artery clamping, using hemostatic agents and selective suturing when necessary. Perioperative, functional, and oncologic outcomes were compared with 313 patients who underwent standard RAPN with parenchymal suturing. **Results**: The median operative time was 110 min (IQR 100–140), and the median estimated blood loss was 180 mL (IQR 100–250). The overall complication rate was 8.4%, predominantly Clavien–Dindo grade I–II, with no conversions to open surgery. The median decline in estimated glomerular filtration rate (eGFR) at three months was 5.5% (IQR 3.5–8.9; *p* = 0.56), and no cases of acute kidney injury were recorded. The positive surgical margin rate was 3.7%, and no tumor recurrences were observed during the 12-month follow-up period. **Conclusions**: Off-clamp, sutureless or selectively sutured robotic-assisted partial nephrectomy (RAPN) was not associated with increased perioperative risk, renal functional decline, or compromised short-term oncologic control compared with conventional sutured RAPN. These findings indicate that the technique is feasible and safe in appropriately selected patients, although prospective studies with longer follow-up are needed to confirm long-term outcomes and refine patient selection criteria.

## 1. Introduction

Renal cell carcinoma (RCC) represents a significant health burden worldwide, accounting for approximately 434,000 new cases and 180,000 deaths annually [1].

In Europe, RCC incidence continues to rise, with Italy reporting ~13,600 new diagnoses and over 4500 deaths per year [2].

Given the increasing detection of small renal masses through cross-sectional imaging, emphasis has shifted toward nephron-sparing strategies that provide durable oncologic control while preserving renal function [3].

Partial nephrectomy (PN) remains the gold standard for managing localized (≤7 cm, T1 stage) renal tumors, as it ensures oncologic efficacy comparable to radical nephrectomy while minimizing the risk of chronic kidney disease (CKD) [4,5,6].

The introduction of robot-assisted PN (RAPN) has further enhanced the precision of tumor excision and renorrhaphy, reducing perioperative morbidity and expanding indications for minimally invasive nephron-sparing surgery [7].

Traditionally, PN involves temporary clamping of the renal artery to provide a bloodless field during tumor excision and parenchymal reconstruction. However, renal ischemia and mechanical suturing can cause nephron loss and microvascular injury, contributing to postoperative functional decline [8].

Even mild reductions in glomerular filtration rate have been associated with increased cardiovascular morbidity, hospitalizations, and all-cause mortality, underscoring the long-term importance of functional preservation [9,10,11].

These findings have driven efforts to develop off-clamp, selective-clamp, and sutureless or minimally sutured techniques, aiming to reduce ischemic and parenchymal injury [10,11,12,13].

Several single-center and early multicenter studies have reported that off-clamp and sutureless approaches may offer comparable oncologic control with shorter ischemia times, less parenchymal loss, and better short-term renal function compared to conventional clamped PN [12,13,14,15].

Nonetheless, most of these studies were limited by modest sample size, heterogeneous surgical techniques, and short follow-up. Thus, the true impact and reproducibility of on-demand selective suturing during clampless RAPN in broader, high-volume clinical settings remain insufficiently defined.

The present multicentric study was therefore designed to assess perioperative outcomes, renal functional preservation, and oncologic safety associated with off-clamp robot-assisted partial nephrectomy using on-demand selective suturing across several high-volume Italian institutions. This large collaborative experience aims to validate the feasibility and reliability of this approach and clarify its role within contemporary nephron-sparing surgery.

## 2. Materials and Methods

### 2.1. Study Design and Patient Selection

This study is a retrospective review of prospectively collected data from four high-volume centers specializing in robot-assisted partial nephrectomy (RAPN). We included 250 consecutive patients who underwent an off-clamp, sutureless or selectively sutured RAPN for T1 renal tumors (Group 1) between January 2018 and December 2024. All tumors were evaluated preoperatively using both the RENAL and PADUA nephrometry scoring systems to quantify anatomical complexity.

**Figure 1:**
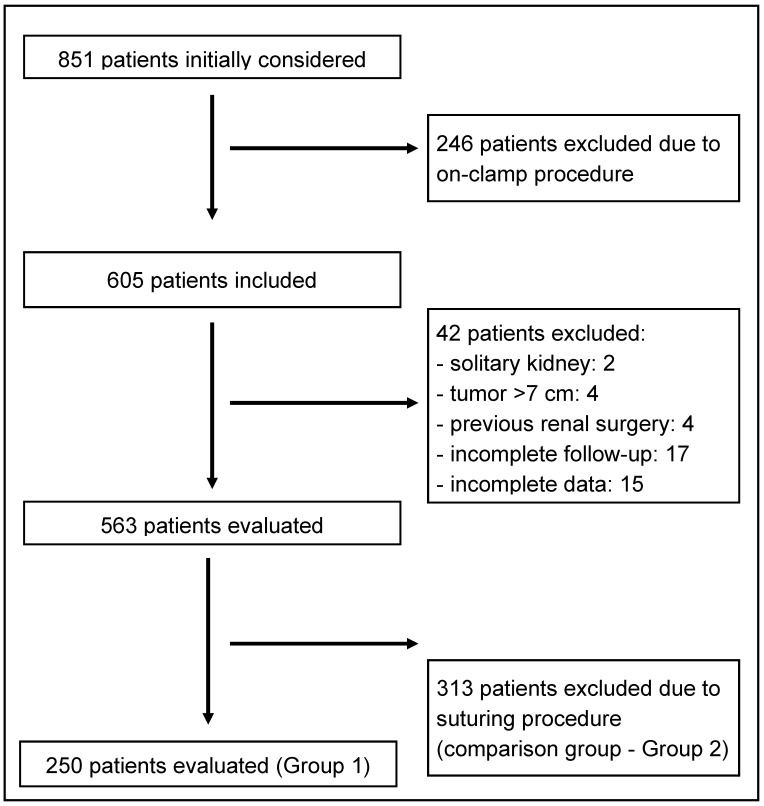
Flowchart.

For comparison, we analyzed data from 313 consecutive patients from the same database, matched for baseline characteristics, including RENAL and Padua scores, who underwent off-clamp RAPN with parenchymal suturing for hemostasis after tumor excision (Group 2) (Figure 1).

### 2.2. Inclusion/Exclusion Criteria

Inclusion Criteria:

- Age ≥ 18 years.
- Localized, clinical T1 (≤7 cm) renal mass.
- Undergoing off-clamp RAPN (sutureless or selectively sutured).
- Availability of preoperative imaging and postoperative renal function data.
- Preoperative RENAL and PADUA nephrometry scoring.

Exclusion Criteria:

- Solitary kidney.
- Tumor > 7 cm.
- Previous renal surgery.
- Missing clinical or follow-up data.

#### 2.2.1. Renal Function Assessment

Renal function was evaluated preoperatively, at hospital discharge, and at the most recent available follow-up (3 and 12 months postoperatively). Serum creatinine values were obtained from institutional laboratory records and used to calculate the estimated glomerular filtration rate (eGFR) according to the Chronic Kidney Disease Epidemiology Collaboration (CKD-EPI) equation [16].

Renal function preservation was defined as the percentage of postoperative eGFR relative to the preoperative baseline value, expressed as:

$$Renal\;function\;preservation\;(\%)=\left(\frac{\mathrm{Postoperative}\;\mathrm{eGFR}}{\mathrm{Preoperative}\;\mathrm{eGFR}}\right)\times 100$$

The percentage decline in eGFR was calculated as:

$$eGFR\;decline\;(\%)=\left(\frac{\mathrm{Preoperative}\;\mathrm{eGFR}-\mathrm{Postoperative}\;\mathrm{eGFR}}{\mathrm{Preoperative}\;\mathrm{eGFR}}\right)\times 100$$

For uniformity, when multiple postoperative measurements were available, the value at 3 months was considered the primary time point for functional analysis, as it reflects the early stabilized renal recovery phase after partial nephrectomy [11].

Long-term renal function (12 months) was analyzed descriptively to assess sustained preservation.

#### 2.2.2. Patients’ Comorbidities

Patient comorbidities were systematically recorded at baseline using preoperative clinical evaluations and electronic medical records.

The following comorbid conditions were specifically assessed:

- Metabolic and vascular: diabetes mellitus, hypertension, and dyslipidemia;
- Cardiovascular: prior myocardial infarction, ischemic heart disease, arrhythmias, or heart failure;
- Hematologic and renal: chronic anemia, coagulation disorders, or preexisting chronic kidney disease (CKD stage ≥ 3);
- Surgical history: previous abdominal or retroperitoneal operations.

These data were prospectively entered into the institutional RAPN databases at each participating center and retrospectively reviewed for analysis. Patients’ comorbidity burden was used for descriptive comparisons and to ensure adequate matching between groups.

Preoperative use of antiplatelet (aspirin, clopidogrel) and anticoagulant therapy (Warfarin or direct oral anticoagulants [DOACs]) was documented for all patients.

Following institutional protocols, antiplatelet agents were discontinued 5–7 days before surgery, and Warfarin or DOACs were withdrawn 3–5 days prior, depending on drug half-life and thromboembolic risk.

Bridging anticoagulation with low-molecular-weight heparin (LMWH) was used selectively in patients with high thrombotic risk.

All medications were restarted postoperatively once hemostasis was secured and renal function was stable.

All patients were followed at 1, 3, 6, and 12 months after surgery through outpatient evaluations, serum creatinine measurement, and imaging when indicated.

#### 2.2.3. Preoperative Tumor Anatomical Complexity

Preoperative tumor anatomical complexity was evaluated using two validated nephrometry systems: the RENAL nephrometry score, as described by Kutikov and Uzzo [17], and the PADUA (Preoperative Aspects and Dimensions Used for an Anatomical) classification, developed by Ficarra et al. [18].

#### 2.2.4. Postoperative Complications

Postoperative complications were categorized according to the Clavien–Dindo classification system [19], which grades surgical complications based on the therapeutic interventions required.

Hospital records were reviewed to identify any readmissions or emergency department visits within 12 months postoperatively, regardless of cause.

All-cause hospitalizations were classified as surgery-related (e.g., hemorrhage, urinary fistula, wound complications) or unrelated (e.g., cardiovascular, respiratory, or infectious events).

Postoperative infections were recorded and classified as urinary tract infection (UTI), wound infection, or systemic infection (sepsis) according to CDC criteria. Only infections occurring during the index hospitalization or within 30 days after surgery were included in this analysis.

#### 2.2.5. Surgical Technique

All RAPN procedures were conducted using the da Vinci Si or Xi robotic platforms (Intuitive Surgical, Sunnyvale, CA, USA). The choice between a transperitoneal or retroperitoneal approach was individualized, based on tumor location, surgeon preference and expertise, and patient body habitus. Pneumoperitoneum was established using the AirSeal^®^ system, with intra-abdominal pressure maintained consistently at 12 mmHg.

At the beginning of each procedure, the renal hilum was identified and eventually isolated to enable prompt vascular control if necessary; however, arterial clamping was deliberately avoided to preserve the clampless technique. After tumor identification, intraoperative ultrasound was routinely utilized to delineate tumor margins and assess depth.

Tumor excision was performed using cold scissors or monopolar curved scissors, while bipolar energy via prograsp forceps was used judiciously to achieve targeted hemostasis. Depending on tumor characteristics, either pure enucleation was performed for well-demarcated lesions or enucleoresection was selected when a rim of normal parenchyma was deemed oncologically prudent.

Topical hemostatic agents, including oxidized regenerated cellulose, gelatin matrix, and fibrin glue, were systematically applied to the resection bed to promote coagulation and minimize residual bleeding. Eventually, hemostasis was optimized through the careful identification and selective ligation of visible arterial or venous branches using 3–0 or 4–0 absorbable sutures.

Renorrhaphy was eventually performed selectively, guided by the intraoperative assessment of the size and depth of the parenchymal defect and any entry into the collecting system. When indicated, a single-layer closure of the renal defect was accomplished using barbed suture to reapproximate tissue without inducing ischemia. Any collecting system breaches were repaired using running 4–0 absorbable sutures.

At the conclusion of the procedure, a closed-suction drain was routinely placed in the renal fossa to monitor for postoperative bleeding or urinary leakage.

The decision to perform the on-demand selective suturing technique was based on emerging scientific evidence and the surgeons’ evidence-based experience. This approach was initially focused on cases where it could be applied most easily (such as small, very exophytic, poorly vascularized lesions) and was then applied more widely.

#### 2.2.6. Data Collection and Outcomes

We gathered data on demographic, clinical, and perioperative characteristics. Anatomic tumor complexity was assessed using both the RENAL and PADUA nephrometry scores. The primary outcomes assessed were intraoperative blood loss, operative time, and complication rates classified by the Clavien–Dindo system. Secondary outcomes included preservation of renal function—assessed by serum creatinine levels and estimated glomerular filtration rate (eGFR)—and oncologic outcomes at a 12-month follow-up.

#### 2.2.7. Statistical Analysis

All statistical analyses were performed using IBM SPSS Statistics, version 29.0 (IBM Corp., Armonk, NY, USA).

All datasets were reviewed for completeness. Cases with missing essential data (median tumor size, baseline renal function, or follow-up eGFR) were excluded from the analysis. For variables with <5% missing values, pairwise deletion was applied. No variable exceeded this threshold.

Patients in Group 1 were matched 1:1 with those in Group 2 using propensity score matching (PSM) based on age, sex, median tumor size, baseline eGFR, and RENAL and PADUA scores [20]. Propensity scores were generated via logistic regression, and nearest-neighbor matching without replacement was applied using a caliper width of 0.2 of the standard deviation of the logit of the propensity score. Post-matching balance was verified by comparing standardized mean differences (SMD), with an SMD < 0.1 considered indicative of adequate balance across covariates.

Continuous variables were assessed for normality using the Shapiro–Wilk test and visual inspection of Q–Q plots, with homogeneity of variances verified using Levene’s test. Normally distributed data are presented as mean ± standard deviation (SD) and compared using Student’s *t*-test, while non-normally distributed data are presented as median (interquartile range, IQR) and compared using the Mann–Whitney U test. Categorical variables are reported as frequencies and percentages and analyzed using the χ^2^ test or Fisher’s exact test, as appropriate.

Univariate analyses were initially performed to identify variables associated with key outcomes (operative time > median, presence of complications ≥ Clavien–Dindo grade II, positive surgical margins, and >20% postoperative eGFR decline). Significant predictors (*p* < 0.10) were entered into a multivariate logistic regression model to identify independent predictors of these outcomes [21]. Multicollinearity among predictors was assessed using the variance inflation factor (VIF), with VIF > 2.5 considered indicative of problematic collinearity.

For key continuous outcomes, we provided 95% confidence intervals for the mean; when only median and IQR were available, SD was approximated as IQR/1.349, and when only the range was available, SD was approximated as (max-min)/4; the reported median was used as a mean proxy solely for CI construction, given the near-symmetric distributions. We conducted all analyses using the statistical software STATA/SE version 18 (StataCorp, College Station, TX, USA). Statistical significance was set at *p* < 0.05 (two-sided). Details of the statistical methodology are provided in Appendix A.

## 3. Results

A total of 250 patients who underwent off-clamp, sutureless, or selectively sutured robotic-assisted partial nephrectomy (RAPN) (Group 1) were included. The median age was 58 years (IQR 52–67), with 58% males and 42% females. The median tumor size was 3.2 cm (IQR 2.4–4.5).

Baseline demographic and clinical characteristics were comparable between groups, with no statistically significant differences in age (median 58 vs. 59 years, *p* = 0.28), tumor size (median 3.2 vs. 3.3 cm, *p* = 0.42), or other evaluated parameters (Table 1).

The prevalence of hypertension, diabetes mellitus, and cardiovascular disease (including prior myocardial infarction, ischemic heart disease, or arrhythmias) did not differ significantly between cohorts. Similarly, there were no differences in chronic kidney disease (CKD ≥ stage 3), hematologic disorders, or previous abdominal operations.

The overall comorbidity burden, expressed as the Charlson Comorbidity Index (CCI), was well balanced across groups (median 2 [IQR 1–3] vs. 2 [IQR 1–3]; *p* = 0.74). These findings confirm that the two populations were similar in terms of systemic health status and surgical risk at baseline.

The prevalence of preoperative antithrombotic therapy was comparable between groups 2 (Table 2). In Group 1, 22 patients (8.8%) were receiving antiplatelet agents and 10 (4.0%) were on chronic anticoagulation (Warfarin or DOACs), compared to 28 (8.9%) and 13 (4.2%) in Group 2 (*p* = 0.96 and *p* = 0.89, respectively). All agents were discontinued per institutional protocol, and no perioperative bleeding complications were attributed to antithrombotic management.

Tumor complexity, assessed by both the RENAL and PADUA nephrometry scoring systems, was comparable between groups (Table 3).

For the RENAL score, the proportions of low-, moderate-, and high-complexity tumors did not differ significantly (38.4% vs. 37.4%; 50.8% vs. 51.4%; 10.8% vs. 11.2%; *p* = 0.82), with a median score of 7 (IQR 6–8) in both groups (*p* = 0.94). Similarly, the PADUA score distribution was similar (low 28.4% vs. 29.1%; intermediate 55.6% vs. 55.0%; high 16.0% vs. 16.0%; *p* = 0.87), with identical median values of 8 (IQR 7–9) (*p* = 0.91). These findings confirm that both cohorts were comparable in preoperative anatomical complexity.

In Group 1, the median operative time was 118 min (IQR 100–140) and median estimated blood loss was 150 mL (IQR 100–250). The overall complication rate was 8.4% (95% CI 5.6–12.5) in Group 1 and 9.6% (95% CI 6.8–13.4) in Group 2 (*p* = 0.61). Conversion to open surgery occurred in 0.0% (95% CI 0.0–1.5) vs. 0.6% (95% CI 0.2–2.3), respectively (*p* = 0.21). Perioperative and hospital-acquired infections were uncommon in both groups. Urinary tract infections occurred in 6 patients (2.4%) in Group 1 and 8 (2.6%) in Group 2 (*p* = 0.89), all managed successfully with antibiotics. No wound infections, abscesses, or sepsis events were reported. These findings indicate that both surgical techniques were associated with a low perioperative infectious risk (Table 4).

At 3 months, the median decline in estimated glomerular filtration rate (eGFR) was 5.6% (IQR 3.4–9.2) in Group 1 and 5.8% (IQR 3.7–8.9) in Group 2 (*p* = 0.56). No cases of acute kidney injury were recorded in Group 1 (0.0%, 95% CI 0.0–1.5), while one case occurred in Group 2 (0.3%, 95% CI 0.1–1.8).

The positive surgical margin (PSM) rate was 3.6% (95% CI 1.9–6.7) in Group 1 and 3.5% (95% CI 2.0–6.2) in Group 2 (*p* = 0.89). At 12 months, no tumor recurrence was detected in Group 1 (0.0%, 95% CI 0.0–1.5), and one recurrence was observed in Group 2 (0.3%, 95% CI 0.1–1.8) (*p* = 0.34). No deaths were reported during follow-up, corresponding to a 100% overall survival rate in both groups.

Multivariate logistic regression analysis (Table 5) was performed to identify independent predictors of perioperative complications and ≥10% postoperative eGFR decline. Variables with *p* < 0.10 in univariate testing were included: surgical technique, age, sex, tumor size, RENAL score, and baseline eGFR.

In multivariate analysis, neither surgical approach (off-clamp vs. conventional) nor demographic factors (age, sex) were independently associated with perioperative complications or renal functional decline. Larger tumor size (OR 1.42, 95% CI 1.08–1.87; *p* = 0.01) and higher baseline eGFR (OR 1.03, 95% CI 1.01–1.06; *p* = 0.02) were independent predictors of a ≥10% eGFR decrease at 3 months. No variable was independently associated with postoperative complications. Due to the low number of oncologic events (PSMs and recurrences), multivariate modeling for oncologic outcomes was not performed.

These findings indicate that the off-clamp, sutureless/selectively sutured RAPN technique was not independently associated with increased perioperative risk or short-term renal functional decline compared with conventional sutured RAPN.

## 4. Discussion

This study presents a comprehensive evaluation of off-clamp RAPN based on a large multicenter Italian cohort. Our findings support the feasibility and short-term safety of the off-clamp approach in managing renal masses, with oncologic outcomes comparable to the conventional technique.

### 4.1. Oncological and Functional Outcomes

The off-clamp, sutureless or selectively sutured RAPN approach achieved oncologic results equivalent to the conventional sutured technique, with both groups showing low and comparable positive surgical margin rates (3.7% vs. 3.5%), underscoring the oncologic safety of ischemia-free partial nephrectomy. This approach enables precise tumor excision while preserving uninvolved renal parenchyma, which is essential for maintaining long-term renal function—particularly critical in patients with solitary kidneys or chronic kidney disease.

In our cohort, the decline in eGFR was comparable between the off-clamp and conventional RAPN groups, indicating no significant functional difference. This finding aligns with previous multicenter studies suggesting that the benefit of ischemia avoidance on renal function remains inconclusive, as postoperative renal recovery often depends more on preserved parenchymal volume than on clamping strategy [18,22], as reported by Khalifeh et al. [22] in one of the largest comparative analyses of off-clamp vs. on-clamp RAPN (they found no significant difference in renal function preservation after controlling for baseline factors) and by Ficarra et al. in a comprehensive review of partial nephrectomy outcomes (they concluded that long-term renal function was primarily determined by the amount of preserved parenchyma rather than ischemia time alone) [18].

When compared with traditional RAPN involving hilar clamping, prior studies—such as those by Simone et al. [23] and Gill et al. [24]—have shown more significant postoperative functional deterioration due to ischemia–reperfusion injury. Our results align with the findings of Porpiglia et al. [25], who demonstrated superior renal function preservation with off-clamp techniques without compromising oncologic safety.

### 4.2. Advantages of the Off-Clamp Approach

In our multicenter cohort, both surgical strategies—off-clamp, sutureless or selectively sutured RAPN and conventional sutured RAPN—demonstrated comparable perioperative and functional outcomes, with low complication rates and excellent short-term renal preservation.

The overall complication rate remained below 10% in both groups, predominantly consisting of minor (Clavien–Dindo grade I–II) events, and no conversions to open surgery were required.

Similarly, postoperative renal function was well preserved, with a modest and equivalent median eGFR decline of approximately 5.5% at 3 months, and no cases of acute kidney injury were observed.

These results reinforce the feasibility and safety of the off-clamp approach, particularly in anatomically suitable cases, while confirming that it does not compromise renal recovery or perioperative outcomes compared with the conventional technique.

Nguyen et al. [26] emphasized that warm ischemia exceeding 25 min significantly affects renal function, while Farinha et al. [27] showed improved outcomes using an off-clamp approach—results echoed by our findings. Furthermore, Bertolo et al. [28] reported enhanced early renal recovery following off-clamp RAPN, without increased risk of complications.

The off-clamp, sutureless, or selectively sutured technique was associated with comparable operative times and blood loss relative to conventional RAPN, while avoiding arterial clamping and parenchymal renorrhaphy in selected cases, which may facilitate the procedure in appropriately selected patients, although this was not formally assessed.

### 4.3. Selective Suturing and Hemostasis

Selective suturing offers a balance between effective hemostasis and minimal parenchymal disruption. By limiting the extent of renorrhaphy, this approach reduces mechanical compression, ischemic injury, and subsequent fibrosis, thereby preserving tissue integrity and functional parenchyma. In our series, we observed low rates of complications such as hemorrhage and urinary fistula, confirming the safety and hemostatic adequacy of this technique.

These results are consistent with previous studies by Buffi et al. [29] and Huang et al. [30], who demonstrated that excessive parenchymal suturing may induce local ischemia and necrosis, potentially contributing to long-term functional deterioration. Our findings further highlight that larger tumor size and higher baseline eGFR, rather than the surgical technique itself, were independent predictors of postoperative renal function decline. The observed association between higher baseline eGFR and greater odds of postoperative eGFR reduction likely reflects a relative change phenomenon—patients with better preoperative renal function have greater measurable declines despite retaining satisfactory absolute function postoperatively.

Collectively, these results reinforce that minimizing suturing and ischemic manipulation can optimize perioperative and functional outcomes in robot-assisted partial nephrectomy, while patient and tumor characteristics remain the primary determinants of postoperative renal recovery.

### 4.4. Perioperative Safety and Complications

The perioperative outcomes affirm that selective suturing during RAPN is both safe and effective. The complication rate remained low, and no significant increase in intraoperative blood loss was observed, indicating reliable hemostasis without the need for extensive sutures. Additionally, the low incidence of urine leakage and reinterventions further supports the safety profile of this technique. Our findings are consistent with those of Pandolfo et al. [31], who demonstrated that selective suturing reduces operative time and improves hemostatic control without increasing adverse events. Similarly, Minervini et al. [32] noted that refined suturing techniques can decrease urinary leak rates while preserving renal function.

Although intraoperative blood loss was comparable between groups, it is plausible that tumor location (cortical vs. medullary) may influence bleeding risk due to regional variations in renal vascularization.

This factor is partly captured by the nephrometry scores used (RENAL and PADUA), which consider the exophytic/endophytic nature and sinus proximity of the lesion.

However, a dedicated analysis stratified by tumor depth was not performed in this study and represents a valuable direction for future research, as it may help refine patient selection and intraoperative planning for off-clamp procedures.

### 4.5. Limitations and Future Directions

This study supports off-clamp and sutureless/selectively sutured RAPN as a viable nephron-sparing approach for small renal tumors, demonstrating favorable perioperative, functional, and short-term oncologic outcomes. However, several limitations must be acknowledged.

First, the retrospective nature of the analysis introduces potential selection and reporting biases. Although the datasets were derived from prospectively maintained institutional databases, the adoption of the off-clamp, sutureless, or selectively sutured technique evolved progressively over time across participating centers. Early in the study period, surgeons tended to apply this approach primarily to cases with more favorable anatomy—typically smaller, exophytic, or peripherally located tumors. With increasing experience and growing confidence in intraoperative hemostatic control, the technique was gradually extended to more complex lesions. This evolution in case selection likely reflects a learning curve effect and may partially explain the low overall complication rates observed. Consequently, while propensity score matching helped balance key preoperative factors, residual confounding due to unmeasured variables cannot be excluded.

Second, the limitations of data capture must be considered. Postoperative complications were recorded from institutional databases and inpatient hospital records, following the Clavien–Dindo classification. However, minor or delayed complications that occurred after discharge—such as low-grade infections, transient hematuria, or small perirenal collections—may not have been consistently reported, particularly if patients sought care at external facilities. Despite standardized follow-up visits and laboratory monitoring at 1, 3, 6, and 12 months, the absence of centralized postoperative surveillance introduces the possibility of underestimation of true complication rates. Accordingly, the low incidence of postoperative adverse events should be interpreted conservatively.

Third, this study was not an intention-to-treat analysis, and unrecognized institutional or surgeon-specific factors—such as evolving perioperative management protocols, differences in intraoperative energy use, or subtle variations in renorrhaphy technique—may have influenced outcomes. The multicenter design, while strengthening external validity and generalizability, inherently introduces heterogeneity in surgical practice and postoperative care pathways, which cannot be fully standardized in retrospective settings.

Finally, the relatively short follow-up period limits conclusions regarding long-term oncologic control and renal functional preservation. While no tumor recurrences were observed at 12 months, longer surveillance is essential to assess durable outcomes, particularly in cases with high-complexity lesions or positive surgical margins.

Future research should focus on prospective, multicenter registries with uniform inclusion criteria, standardized postoperative monitoring (including post-discharge complication tracking), and extended oncologic follow-up. Such initiatives will be critical to validate these findings, clarify the long-term implications of sutureless or selectively sutured off-clamp RAPN, and further refine patient selection criteria to optimize the balance between oncologic safety and maximal nephron preservation.

## 5. Conclusions

In this large multicenter analysis, the off-clamp, sutureless or selectively sutured RAPN approach appeared to be a safe and effective technique for managing T1 renal masses. The absence of significant differences in perioperative, functional, and oncologic outcomes compared with conventional RAPN supports its role as a viable alternative. Notably, the off-clamp strategy consistently preserved renal function similarly to the conventional approach, with minimal eGFR decline and no acute kidney injury, while maintaining low complication rates and favorable oncologic control.

Importantly, multivariate analysis identified larger tumor size and higher baseline eGFR as independent predictors of postoperative renal functional decline, underscoring the influence of patient and tumor characteristics on renal recovery even in the context of ischemia-free surgery. These findings highlight the value of individualized, nephron-sparing strategies tailored to tumor complexity and preoperative renal reserve. Further prospective studies are warranted to validate these results and explore their applicability in more complex renal tumors.

Declaration of generative AI and AI-assisted technologies in the writing process.

During the preparation of this work the authors used ChatGPT 5 in order to improve the readability and language of the manuscript. After using this tool, the authors reviewed and edited the content as needed and take full responsibility for the content of the published article.

## Figures and Tables

**Table 1:** Patients characteristics.

Variable	Group 1 (n = 250)	95% CI (%)	Group 2 (n = 313)	95% CI (%)	*p*-Value
Demographics					
Median age, years (range)	58 (35–80)	52 to 67	59 (36–82)	54 to 68	0.28
Male sex, n (%)	145 (58%)		184 (59%)		0.87
Mean tumour size (cm)	3.2 (1.7–5.8)	2.4 to 4.5	3.3 (1.9–6.1)	2.5 to 4.1	0.42
Perioperative outcomes					
Operative time (min), median (range)	118 (100–140)	114.3 to 121.7	120 (105–145)	116.7 to 123.3	0.33
Estimated blood loss, mL, median (range)	150 (50–400)	139.2 to 160.8	160 (60–410)	150.3 to 169.7	0.47
Complication rate, n (%)	21 (8.4%)		30 (9.6%)		0.61
Conversion to open, n (%)	0 (0%)		2 (0.6%)		0.21
Functional outcomes					
Median eGFR decline at 3 months (%)	5.5%	3.4–9.2	5.8%	3.7–8.9	0.56
Acute kidney injury, n (%)	0 (0%)		1 (0.3%)		0.37
Oncologic outcomes					
Positive surgical margins, n (%)	9 (3.7%)		11 (3.5%)		0.89
Recurrence at 12 months, n (%)	0 (0%)		1 (0.3%)		0.34

eGFR: estimated glomerular function rate, IQR: interquartile range.

**Table 2:** Baseline comorbidities of the study population.

Variable	Group 1 (n = 250)	Group 2 (n = 313)	*p*-Value
Hypertension	124 (49.6%)	159 (50.8%)	0.78
Diabetes mellitus	38 (15.2%)	49 (15.7%)	0.88
Cardiovascular disease	41 (16.4%)	50 (16.0%)	0.92
CKD (≥stage 3)	12 (4.8%)	17 (5.4%)	0.77
Hematologic disorder	6 (2.4%)	7 (2.2%)	0.89
Previous abdominal surgery	58 (23.2%)	74 (23.6%)	0.94
CCI, median (IQR)	2 (1–3)	2 (1–3)	0.74

CCI: Charlson Comorbidity Index; CKD: chronic kidney disease.

**Table 3:** Nephrometry score distribution.

**RENAL Nephrometry Score Distribution by Group**			
RENAL Score Category	Group 1 (n = 250)	Group 2 (n = 313)	*p*-value
Low (4–6)	96 (38.4%)	117 (37.4%)	0.82
Moderate (7–9)	127 (50.8%)	161 (51.4%)	
High (10–12)	27 (10.8%)	35 (11.2%)	
Median RENAL score (IQR)	7 (6–8)	7 (6–8)	0.94
**PADUA Nephrometry Score Distribution by Group**			
PADUA Score Category	Group 1 (n = 250)	Group 2 (n = 313)	*p*-value
Low (6–7)	71 (28.4%)	91 (29.1%)	0.87
Intermediate (8–9)	139 (55.6%)	172 (55.0%)	
High (≥10)	40 (16.0%)	50 (16.0%)	
Median PADUA score (IQR)	8 (7–9)	8 (7–9)	0.91

**Table 4:** Perioperative and hospital-acquired infection rates.

Variable	Group 1 (n = 250)	Group 2 (n = 313)	*p*-Value
Urinary tract infection	6 (2.4%)	8 (2.6%)	0.89
Wound infection	0 (0%)	0 (0%)	–
Sepsis/deep infection	0 (0%)	0 (0%)	–

**Table 5:** Multivariate Logistic Regression Predicting Perioperative Complications and ≥10% eGFR Decline.

Variable	OR (Complications)	*p*-Value	OR (≥10% eGFR Decline)	*p*-Value
Group 1 (vs. Group 2)	0.89 (0.48–1.63)	0.69	0.95 (0.61–1.49)	0.82
Age (per year)	1.01 (0.98–1.04)	0.41	1.02 (0.99–1.05)	0.18
Male sex	1.23 (0.68–2.24)	0.49	1.11 (0.71–1.76)	0.64
Median Tumor size (per cm)	1.10 (0.86–1.42)	0.43	1.42 (1.08–1.87)	0.01 *
RENAL score	1.06 (0.88–1.28)	0.55	1.15 (0.97–1.37)	0.10
Baseline eGFR (per unit)	0.99 (0.97–1.01)	0.25	1.03 (1.01–1.06)	0.02 *

* indicates statistical significance (*p* < 0.05).

## Data Availability

Data collection was conducted in accordance with the World Medical Association Declaration of Helsinki. The standard of care remained consistent with that provided according to international guidelines for patients participating in the study. All participants were required to provide written informed consent.

## Appendix A. Statistical Analysis Summary

- Study Design: Retrospective comparative analysis of prospectively collected multicenter data.
- Matching: 1:1 comparison between off-clamp RAPN (Group 1) and conventional RAPN with parenchymal suturing (Group 2), based on comparable baseline characteristics.
- Descriptive Statistics:
  ○ Continuous variables expressed as median (interquartile range [IQR]).
  ○ Categorical variables presented as count (percentage).
- Comparative Tests:
  ○ Continuous variables: Student’s *t*-test or Mann–Whitney U test as appropriate.
  ○ Categorical variables: Chi-square (χ^2^) or Fisher’s exact test.
- Regression Analysis:
  ○ Univariate analysis performed using Pearson χ^2^ test.
  ○ Multivariate logistic regression conducted to identify predictors of ypT0 (only relevant for broader study context) [14,15].
- Statistical Significance:
  ○ Defined as *p* < 0.05 for all comparisons.
